# The impact of the COVID- 19 pandemic on women’s mental health: empirical evidence from Vietnam

**DOI:** 10.1186/s41155-025-00349-y

**Published:** 2025-04-14

**Authors:** Nguyen Thi To Vy, Luong Vinh Quoc Duy

**Affiliations:** University of Economics Ho Chi Minh City (UEH), Ho Chi Minh City, Vietnam

**Keywords:** Women, Mental health, Pandemic, WHO- 5, OECD- 7, Developing countries

## Abstract

**Background:**

Women’s mental health has emerged as a growing concern amid the COVID- 19 as studies show women are more likely to suffer symptoms of depression and anxiety than men. Social and economic disadvantages are believed to correlate with women’s mental health during the pandemic. However, studies mainly show the prevalence of mental health problems among women during COVID- 19 across the globe while the covariates are not extensively reported. Moreover, the literature on mental health is believed to be overlooked in the developing countries.

**Objective:**

This study aims to investigate women’s mental health in Vietnam under the context of COVID- 19.

**Methods:**

This study used a novel data set from our survey of women located in five provinces and cities in Vietnam. The study sample included 519 observations. Women’s mental health is measured by WHO- 5 and OECD- 7, these two inventories are designed to measure the subjective wellbeing of respondents. Our research utilized correlational analysis to explore the connections between women’s mental wellbeing and their social and economic characteristics.

**Results:**

Results indicate statistically significant associations between women’s mental wellbeing and their personal characteristics such as education level, employment status, income, age, number of children, marital status, and household appliances.

**Conclusion:**

Policymakers should prioritize policies to encourage women to upgrade their education and skills should receive more attention from policymakers, and attention should be particularly placed on women in the vulnerable age groups for the prevention or mitigation of psychological effects from social and economic shocks. As a preventive measure for mental health problems, it is essential to popularize knowledge on mental health and how to cope with the related issues. Also, nationwide data on people’s mental health should be collected regularly to facilitate more studies to provide insights into mental health issues.

## Introduction

Mental health refers to the condition of mental wellbeing that allows individuals to manage their life stress, be aware of their abilities, perform well in study and work, and contribute to the community around them (WHO, 2022). People’s mental health is believed to receive increasing concerns across the globe because of the occurrence of unfavorable events (WHO, 2005). Moreover, gender gap in mental health problems persists as women are reported to be almost twice as much as men to suffer depression (Yu, 2018). Women’s mental health has emerged as a growing concern amid the COVID- 19 as studies show women are more likely to suffer symptoms of depression and anxiety than men (Ettman et al., 2020). Social and economic disadvantages are believed to correlate with women’s mental health during the pandemic (Penninx et al., 2022; Santomauro et al., 2021; Xiong et al., 2020). However, studies on mental health issues in women during the COVID- 19 pandemic is claimed to be scarce (Thibaut & van Wijngaarden-Cremers, 2020). Reports show the prevalence of mental health problems among women during COVID- 19 across the globe, but the covariates are not reported (Basu et al., 2021). In addition, many available studies are found mainly focused on pregnant women or on the context of developed economies (e.g. Ho-Fung et al., 2022; Matvienko-Sikar et al., 2020; Perzow et al., 2021; Salehi et al., 2020).

Nation-wide statistics on mental health problems in Vietnam are limited availability. A brief report from the Ministry of Health Portal (2023) shows the depression rate is 2.45% and the suicide rate is 5.87 per 100,000 population for the year 2014 and 2015 respectively. Although mental health is currently reported not a problem for people in the country, Overseas Development Institute and UNICEF Vietnam (2011) believe it is worth paying more attention to people’s mental health because family and social pressures are the potential risks for mental disorders. It appears that studies on mental health in the country are few and mostly focus on the health care sector. For instance, Tran et al. (2024) examine workplace wellbeing of a group of pharmacy workers, Thuan and Hung (2023) investigate the clinical factors related to depression in patients with ischemic stroke, and Nguyen et al. (2022) study the impact of COVID- 19 on psychological wellbeing of pregnant women. These studies confirm the prevalence of mental health problems, but the social and economic covariates are not reported.

This study aims to investigate women’s mental health in Vietnam under the context of COVID- 19. Contributions of this study are threefold. Firstly, this study contributes to the literature on mental health, the issue believed to be overlooked in the developing countries (Patel, 2007; WHO, 2000). Secondly, this study examines women’s mental health and its social and economic covariates amid the COVID- 19, which were not extensively covered in previous studies. And thirdly, this study uses a sample of general women rather than pregnant women or those in the health care sector as in previous studies. To record the mental health status of women participating in our survey, WHO- 5 and OECD- 7 were employed. These two inventories are designed to measure the subjective wellbeing of respondents (OECD, 2013; WHO, 1998).

## Methodology

### Sample

The survey was conducted between June 2020 and September 2021 across one city and four provinces in Vietnam, in particular: Ho Chi Minh City, Gia Lai, Ha Tinh, Lam Dong, and Bac Ninh. Following the convenience sampling and snowballing approach as described by Ritchie and Lewis (2003), one thousand people were invited to participate in the survey. Some 804 responded, but the sample used for analysis includes 519 observations after removing incomplete or inconsistent returned questionnaires. All respondents have provided their verbal consent to participate in the survey. The questionnaire consists of questions to measure respondents’ mental wellbeing and their social and economic characteristics. The questionnaire was originally written in English language, then it was translated into Vietnamese language by one author and double-checked by the other author following two-way translation. The authors administered the survey with help from local contacts in finding the target respondents. The respondents filled in the questionnaires and posted them to the authors. Where travel was limited due to outbreaks of COVID- 19, the survey was conducted via the telephone or online platforms such as Google Forms, Zoom, or Zalo.

### Measurement

Following WHO (1998), the WHO- 5 inventory was adopted to assess the self-rated mental wellbeing of respondents. This inventory includes five six-point scale items to record respondent’s feeling in the past 2 weeks. The raw score was computed by adding up the figures of the five items, and higher raw score means better wellbeing. Next, the standardized percentage score (SPS), ranging from 0 to 100, was created by multiplying the raw scores with 4 (Topp et al., 2015). In addition, OECD- 7 inventory, which contains five seven-point scale items, was also employed to measure the subjective wellbeing of those participating in the survey. The sum of responses to each of questions reflects respondents’ level of satisfaction with their lives, for instance, the larger sum means higher level of life satisfaction (OECD, 2013). Table 1 presents result of descriptive statistics, reliability, and descriptions of WHO- 5 and OECD- 7 scales. The reliability of the scales was measured by Cronbach’s alpha, and the estimated coefficients are all larger than the threshold of 0.70 as recommended by Hair et al. (2010).

**Table 1:** Descriptive statistics, reliability, and descriptions of WHO- 5 and OECD- 7 scales

	WHO–5 scale				OECD–7 scale							
Description												
No	Items			Scores	Items							**Scores**
1	I feel cheerful and in good spirits			0–5	In most ways, my life is close to my idea							1–7
2	I feel calm and relaxed			0–5	The conditions of my life are excellent							1–7
3	I feel active and vigorous			0–5	I am satisfied with my life							1–7
4	I wake up feeling fresh and rested			0–5	So far, I have gotten the important things that I want in my life							1–7
5	My daily life is filled with things that interest me			0–5	If I could live my life over, I would change almost nothing							1–7
Interpretation of scales												
	1	2	3		1	2	3	4	5	6	7	
Point	^a^SPS ≤ 28	28 < SPS ≤ 50	SPS > 50		5–9	10–14	15–19	20	21–25	26–30	31–35	
Meaning	Depression	Poor mental well-being	Good well-being		Extremely dissatisfied	Dissatisfied	Slightly dissatisfied	Neutral ^b^	Slightly satisfied	Satisfied	Extremely satisfied	
Descriptive statistics												
Average point (min)	2.47				3.49							
Base point^c^	2.50				3.00							
Average point (max)	2.76				3.73							
Gross average point	13.36				18.19							
General base point^d^	12.50				17.00							
Cronbach’s alpha coefficient	0.92				0.86							

^a^*SPS* standardized percentage score

^b^Neutral: neither satisfied nor dissatisfied

^c^Base point of WHO- 5 is the medium of 5 (levels from 0–5); base point of OECD- 7 is the medium of 6 (levels from 1 to 7)

^d^General base point of WHO- 5 is the medium of 25 (levels from 0 to 25); general base point of OECD- 7 is the medium of 34 (levels from 1 to 35)

Source: Authors

## Results

Table 2 presents the summary statistic of qualitative variables used for the analysis. Most of the respondents are in marital status and motherhood, while a small proportion of them are staying single or in unfavorable marital status. Nearly half of the respondents have graduated from a university, and most of them reported having a job, of which around two-thirds are non-farm ones. Around 80% of respondents are living in rural areas, and over half of the sampled women reported earning five million VND per month or less. Almost all the respondents reported owning a house or an apartment, and over half of them are younger than 40 years old.

**Table 2:** Summary statistics of qualitative variables

Qualitative variables	Definition/explanation	Proportion (%)
Marital status		
Married	1: if the respondent is married	85.74
Single	2: if the respondent is single	7.90
Unfavorable	3: if the respondent is at the other status (widowed, divorced, or separated)	6.36
Education		
High school or lower	1: if the respondent completed high school or lower level	55.49
University	2: if the respondent has a university degree	44.51
Types of job		
Non-job	1: if the respondent is unemployment, or disable, or a student	3.28
Agricultural job	2: if the respondent works as a farmer	26.20
Formal job	3: if the respondent has a full-time non-farm job	63.01
Informal job	4: if the respondent has a part-time non-farm job or does mainly housework	7.51
Salary		
Five million VND per month or less	1: if the respondent earns five million VND per month or less	56.65
Above five million VND per month	2: if the respondent earns higher than five million VND per month	43.35
Living area		
Rural	1: if the respondent lives in the rural area	80.35
Urban	2: if the respondent lives in the urban area	19.65
House ownership		
Yes	1: if the respondent owns a house/an apartment	94.60
No	0: otherwise	5.40
Ethnic group		
Kinh ethnic	1: if the respondent is Kinh ethnic	95.18
Others	0: otherwise	4.82
Age group		
Younger than 40 years old	1: if the respondent is younger than 40 years old	52.61
40 years old or older	2: if the respondent is 40 years old or older	47.39
Children		
Yes	1: if the respondent has at least one child	88.05
No	0: otherwise	11.95
Children aged from 0 to 14 years		
Yes	1: if the respondent has at least one child aged from zero to 14 years old	54.14
No	0: otherwise	45.86
Children aged from 15 years or older		
Yes	1: if the respondent has at least one child age from 15 years old or older	45.47
No	0: otherwise	54.53

Source: Authors

Table 3 shows the summary statistic of quantitative variables used for the analysis. Respondent’s total number of children aged from 0 to 14 years old is nearly two, while this figure is a bit larger than two for children aged fifteen or older. Total household appliances or devices are used as the proxies for the living conditions of the respondents and their families. Respondent’s total number of household appliances for modern life, by average, is over five, and this figure is around three for the devices that help families to connect with social networks.

**Table 3:** Summary statistics of quantitative variables

Variables	Description	Mean	Standard deviation	Min–max
Total number of children		1.92	1.16	0–8
Aged 0 to 14	Respondent’s total number of children aged from 0 to 14 years old	1.68	0.65	0–6
Aged 15 or older	Respondent’s total number of children aged from 15 years or older	2.22	1.98	0–8
Total household appliances/devices		8.51	3.02	1–15
For modern life	Respondent’s total number of household appliances/devices for modern life (motorbike, car/canoe, air conditioner, refrigerator, microwave oven, electric stove/gas stove/electromagnetic stove, wash machine, generator, heater)	5.35	1.97	1–10
For social network	Respondent’s total number of household appliances/devices that enable social network connection (computer/laptop, smart phone, analog television, and other digital entertainment systems)	3.17	1.32	0–5

Source: Authors

### Marital status

The results from Table 4 suggest that, according to WHO- 5, there is no statistically significant difference in women’s mental wellbeing between those who are married and otherwise, but results from OECD- 7 confirm the difference. This finding is in line with previous studies, which provide mixed results. For example, some studies confirmed the important role of marital status and women’s mental wellbeing amid the COVID- 19 (Pariente et al., 2020; Peng et al., 2022). However, other studies suggested that the relation between women’s mental wellbeing and their marital status may vary according to specific country contexts (Lee & Ono, 2012), or personal preference for age at marriage (Carlson, 2012). Moreover, Fuhrer et al. (1999) claimed that the marital status and women’s mental health was not related.

**Table 4:** Correlation analysis of women’s mental wellbeing among qualitative and quantitative variables

Qualitative variables	WHO- 5		OECD- 7	
	*x*2	Fisher’s exact	*x*2	Fisher’s exact
Marital status	0.42	0.44	0.01***	0.01***
Married and children	**0.34**	**0.34**	0.01***	0.01***
Children	0.17	0.17	0.11	0.11
Children aged from 0 to 14 years	0.00***	0.00***	0.00**	–
Children aged from 15 years or older	0.00***	0.00***	0.00***	–
Education	0.05*	–	0.00***	–
Types of job	0.01**	0.01**	0.01**	–
Salary	0.01**	0.00***	0.24	–
Age group	0.01***	0.01***	0.00***	–
Living area	0.36	0.38	0.03***	0.03***
House ownership	0.18	0.16	0.00***	–
Ethnic group	0.13	0.08*	0.03**	0.01**
Quantitative variables	**WHO- 5**		**OECD- 7**	
	**Prob > *F***		**Prob > *F***	
Age	0.00***		0.00***	
Women aged below 40 years	0.78		0.77	
Women aged over 40 years	0.03**		0.09*	
Total number of children	0.18		0.05*	
Total number of children aged from 0 to 14 years old	0.99		0.40	
Total number of children aged from 15 years or older	0.00***		0.00***	
Total type of household appliance	0.00***		0.00***	
Total type of appliance connecting to modern life	0.00***		0.00***	
Total type of appliance connecting to social network	0.00***		0.00***	

(Note: *N* = 519; *p < 0.1; **p < 0.05; ***p < 0.01)

Source: Authors

### Children

Results from Table 4 show no statistically significant difference in mental wellbeing between women who are raising their children compared to those who are not. However, a look into two subgroups: women with children aged from 0 to 14 years old and those with children aged fifteen or older, the results from WHO- 5 and OECD- 7 show significant disparities in mental wellbeing among women. Similarly, the relation between women’s mental wellbeing and the total number of their children only matters according to results from OECD- 7. But this association is confirmed by outcomes from WHO- 5 and OECD- 7 estimation when sampled women have more children aged from 15 years or older. The interaction between *Married* and *Children* is also examined in this study, and results from OECD- 7 reveal a statistically significant difference between the group of married women raising children and other groups. Findings from related studies are mixed regarding this issue. Vanassche et al. (2013) found a negative satisfaction of life in the group of married women who have older children. While the study by Zimmermann and Easterlin (2006) showed that raising children had no effect on women’s life satisfaction regardless of their marital status.

### Education

Results from WHO- 5 and OECD- 7 both show a statistically significant difference in women’s mental wellbeing across education levels (Table 4). This finding is in line with Raymo and Zhou (2012) who found that higher education and women’s subjective wellbeing was related. Ghosh et al. (2017) confirmed that young married woman, whose education level is equivalent to her husband’s, has higher mental well-being and happiness than others. Other studies also support the relation between schooling time and women’s mental wellbeing (see Mutz, 2021; Ruggeri et al., 2020).

### Employment status and salary level

Women’s mental wellbeing is found to be associated with their job characteristics, and this finding is confirmed by the results from WHO- 5 and OECD- 7 (Table 4). Mental wellbeing is also found to be correlated with women’s salary levels, and this finding is depicted by results from WHO- 5. These findings are similar to that of Ruggeri et al. (2020) who found unemployed individuals tend to exhibit lower level of subjective wellbeing regardless of their gender. Moreover, Kimhi et al. (2020) stressed that the loss of job due to the pandemic worsened people’s subjective wellbeing.

### Living area, private house, and ethnic

Results from WHO–5 scores in Table 4 reveal that women’s mental wellbeing is not statistically significant associated with the location where they are residing, or whether they own a house, or their ethnic group. However, results from the OECD- 7 scores show that women’s mental wellbeing and their living location is a statistically significant correlation. Glaeser et al. (2016) showed that people residing in the big cities may earn much more real salaries, but face a decline in happiness. Rodríguez and Camacho (2024) also conclude that women living in the urban areas are more likely to report higher perceived negative impact on their mental wellbeing compared to their rural counterparts.

### Age

The relation between women’s mental wellbeing and their age was analyzed through qualitative and quantitative proxies. A statistically significant disparity in the mental wellbeing is identified between the group of women younger than 40 years of age and the group of those who are older (the qualitative variables in Table 4). Results from the group of quantitative variables in Table 4 depict a closer picture of the relationship between women’s mental wellbeing and their age. This link is found statistically significant for the sampled women who are over 40 years old, according to OECD- 7 scales. Headey and Wearing (1989) and Ruggeri et al. (2020) observed that older people were less likely to have positive feelings than younger ones.

### Household appliances

Outcomes from analysis of WHO- 5 and OECD- 7 scales suggest that the number of household appliances owned or used by the sampled women are statistically significant correlated with their mental wellbeing (Table 4). This finding also holds for results from the analysis performed separately for the group of appliances for modern life appliances and the group of devices for social network. According to Cai et al. (2022), the possession of household modern appliances and occupants’ self-rated psychological conditions was found to be positively related. Meanwhile, Verduyn et al. (2022) suggested that the connection to social networks might be either positively or negatively associated with the users’ wellbeing.

## Discussion and conclusion

While concerns over women’s mental health amid the pandemic are not new, few studies investigate the link between mental health and women’s social and economic characteristics. This study contributes to literature by examining women’s mental health and the associated covariates during the COVID- 19 using a sample of women residing in one city and four provinces in Vietnam. Women’s mental health was measured by WHO- 5 and OECD- 7, the inventories to record self-rated mental wellbeing of respondents. Women’s mental wellbeing is found to statistically significant related with their educational level, types of job, age, and household appliances. Future studies should extend the scope of survey to increase the representativeness of the sample.

Based on our findings, some policy implications are suggested as follows. Firstly, as women’s mental wellbeing is found to be correlated with their educational level, policies to encourage women to upgrade their education and skills should receive more attention from policymakers. Secondly, attention from policymakers should also be particularly placed on women in the vulnerable age groups for the prevention or mitigation of psychological effects from social and economic shocks. Thirdly, as a preventive measure for mental health problems, it is essential to popularize knowledge on mental health and how to cope with the related issues. Fourthly, policies to improve the quality of women’s jobs and enhance their access to appliances and devices for modern life and network connections should also be of priority. And fifthly, nationwide data on people’s mental health should be collected regularly to facilitate more studies to provide insights into mental health issues.

## Data Availability

The datasets used in the current study are available from the corresponding author on reasonable request after the paper is published.

## Abbreviations

WHO: World Health Organization
OECD: Organization for Economic Cooperation and Development
